# DPP3 promotes breast cancer tumorigenesis by stabilizing FASN and promoting lipid synthesis: DPP3 promotes breast cancer tumorigenesis

**DOI:** 10.3724/abbs.2024054

**Published:** 2024-04-24

**Authors:** Xiaoyu Fu, Xu Li, Weixing Wang, Juanjuan Li

**Affiliations:** 1 Department of Breast and Thyroid Surgery Renmin Hospital of Wuhan University Wuhan 430060 China; 2 Department of General Surgery Renmin Hospital of Wuhan University Wuhan 430060 China; 3 Hubei Key Laboratory of Cell Homeostasis College of Life Sciences TaiKang Center for Life and Medical Sciences Wuhan University Wuhan 430072 China

**Keywords:** dipeptidyl peptidase 3, fatty acid synthetase, breast cancer

## Abstract

DPP3, a dipeptidyl peptidase, participates in a variety of pathophysiological processes. DPP3 is upregulated in cancer and might serve as a key factor in the tumorigenesis and progression of various malignancies. However, its specific role and molecular mechanism are still unknown. In this study, the expression of DPP3 in breast cancer tissues is analyzed using TCGA database. Kaplan-Meier survival analysis is performed to estimate the effect of DPP3 on the survival outcomes. To explore the biological function and mechanisms of DPP3 in breast cancer, biochemical and cell biology assays are conducted *in vitro*. DPP3 expresses at a higher level in breast cancer tissues than that in adjacent tissues in both TCGA database and clinical samples. Patients with high expression of DPP3 have poor survival outcomes. The proliferation and migration abilities of tumor cells with stable *DPP3* knockout in breast cancer cell lines are significantly inhibited, and apoptosis is increased *in vitro*. GSEA analysis shows that DPP3 can affect lipid metabolism and fatty acid synthesis in tumors. Subsequent experiments show that DPP3 could stabilize FASN expression and thus promote fatty acid synthesis in tumor cells. The results of the metabolomic analysis also confirm that DPP3 can affect the content of free fatty acids. This study demonstrates that DPP3 plays a role in the reprogramming of fatty acid metabolism in tumors and is associated with poor prognosis in breast cancer patients. These findings will provide a new therapeutic target for the treatment of breast cancer.

## Introduction

Breast cancer is the most common malignancy among women worldwide and poses a serious threat to women’s health [1]. It is important to deeply explore the molecular mechanism underlying the occurrence and development of breast cancer to develop optimal treatment strategies for breast cancer patients. DPP3, a cytoplasmic peptidase (one of the protease families of EC 3.4.14), is a member of the zinc-dependent M49 metallopeptidase family. DPP3 is widely distributed in various tissues, such as red blood cells, white blood cells, lungs, heart, kidney, intestine, skeletal muscle, skin, brain, liver, and spleen, and partially in soluble form in circulating blood [2]. DPP3 can lyse a variety of bioactive peptides, including angiotensin, bilinogen enterohepatic circulation, enkephalin, endorphin and dipeptidyl derivatives, and is involved in a variety of pathophysiological processes, including blood pressure regulation, inflammation regulation and pain signaling [ 3, 4]. In recent years, emerging studies have revealed that DPP3 is upregulated in aggressive brain glioma [5], ovarian cancer [6], endometrial cancer [7], and bowel cancer [8]. It has been shown that overexpressed DPP3 protein is associated with poor prognosis in patients with bowel cancer. In MCF-7 human breast cancer cells, the binding of DPP3 with KEAP1 stabilizes KEAP1, thereby allowing Nrf-2 to accumulate and locate in the nucleus and affect prognosis through the regulation of apoptosis [9]. The high expression of DPP3 in breast cancer indicates poor prognosis *in silico*; however, its specific role and molecular mechanisms are still not well known [10].

Fatty acid is one of the important energy substances in the body. Fatty acid metabolic reprogramming has recently been considered as a key driver of cancer progression and is deeply involved in the occurrence, development, metastasis and drug resistance of tumors. Several studies have shown that miRNA-328 and CDCP1 (CUB-domain containing protein 1) can promote the occurrence and metastasis of breast cancer by regulating fatty acid β-oxidation (FAO) [ 11, 12]. Fatty acid synthesis is also essential for the distant metastasis of breast cancer and resistance to chemotherapy [13]. Li *et al*. [14] demonstrated that targeting the FASN/HIF1α/SLC7A11 pathway could enhance the sensitivity to sorafenib in HCC cells. Tiong *et al*. [15] showed that targeting the metabolic axis of SREBP-1/Hsa-Mir-497/SCAP/FASN in tumors could reverse the chemo-resistant phenotype in NSCLC cells. Therefore, the reprogramming of fatty acid metabolism is an indispensable part of tumor growth, progression and treatment.

In this study, we found that DPP3 over-expression is highly associated with shortened survival time in breast cancer patients. Through its interaction with fatty acid synthetase (FASN), DPP3 can stabilize FASN, induce the synthesis of fatty acids and thus promote the development of breast cancer. These results suggest that the DPP3-FASN interaction is a novel potential therapeutic target for breast cancer treatment.

## Materials and Methods

### Clinical human breast cancer specimens

Primary breast cancer samples and adjacent normal tissues were collected from breast cancer patients who underwent surgical treatment in the Department of Breast and Thyroid Surgery of Renmin Hospital of Wuhan University (Wuhan, China). None of the patients received any systemic or local treatment for breast cancer before surgery. All the collected samples were transported on dry ice and kept in a –80°C refrigerator. All patients signed the written informed consent forms and the baseline information of the patients is shown in Supplementary Table S1. All procedures involving human participants were performed in accordance with the ethical standards of the institutional and/or national research committee and with the 1964 Declaration of Helsinki and its later amendments or comparable ethical standards. The current work was approved by the Research Ethics Committee of Renmin Hospital of Wuhan University.

### Cells and constructs

MDA-MB-231 (HTB-26), 4T1 (CRL-2539), and MCF-10A cell lines were obtained from American Type Culture Collection (ATCC, Manassas, USA) and were cultured in DMEM (Gibco, Grand Island, USA) or RPMI-1640 medium (Gibco) supplemented with 10% fetal bovine serum (FBS; Gibco) and 1% penicillin-streptomycin in an incubator with 5% CO _2_ at 37°C. To knock out the *DPP3* gene in MDA-MB-231 cells using the CRISPR-Cas9 genomic editing system, two single-guide RNAs (sgRNAs; DNA sequences 5′-GAAGGTGAGAACATCCAGGG-3′ and 5′-AGAAGGGAAGCCCTACTACG-3′) predicted by the sgRNA Designer ( https://portals.broadinstitute.org/gpp/public/analysis-tools/sgrna-design) were synthesized in DNA form, annealed into double strands, treated with T4 polynucleotide kinase, and inserted into the *Bsm*BI-digested lentiCRISPR v2 vector (Addgene, Watertown, USA). The two constructs were transfected into MDA-MB-231 cells and selected with puromycin. A similar strategy was used to construct *DPP3*-knockout 4T1 cells using sgRNAs (DNA sequences 5′-CACCGCCGAGTGTTGTATGCACTG-3′ and 5′-AAACCAGTGCATACAACACTCGGC-3′).

### Lentivirus package

HEK293T cells were transfected with the packaging plasmid pLVX-IRES-ZsGreen1 (TaKaRa, Dalian, China) using Lipofectamine 2000 (Life Technologies, Carlsbad, USA). Forty-eight hours after transfection, the lentiviral supernatant was harvested and filtered through a 0.45-μm filter. The filtrate was utilized for subsequent infection of MDA-MB-231 and 4T1 cells together with 10 μg/mL polybrene (Beyotime, Shanghai, China) in the medium. Stable clones were selected by treatment with 10 μg/mL puromycin for 1 week. The protein levels in stable clones were verified by western blot analysis. All plasmid DNA was validated by sequencing.

### Western blot analysis

The cells were harvested and extracted with NP-40 buffer (50 mM Tris-HCl, pH 7.4, 150 mM NaCl, EDTA, 1% Nonidet P-40, phosphatase inhibitor and protease inhibitor) for SDS-PAGE. Approximately 20 mg of tissues was ground with RIPA lysis buffer (BL504A; Biosharp, Guangzhou, China) (50 mM Tris-HCl, pH 7.4, 150 mM NaCl, 1% Triton X-100, 1% sodium deoxycholate, 0.1% SDS, sodium orthovanadate, sodium fluoride, EDTA and leupeptin). The samples were centrifuged at 12,000 *g* for 10 min at 4°C. The supernatant was transferred to new tubes. The protein concentration was detected using a BCA Protein Assay Kit (Thermo Fisher Scientific, Waltham, USA). The loading buffer was added to the protein lysates and boiled for 10 min at 95°C. The proteins were resolved by SDS-PAGE, electrophoretically transferred to PVDF membranes which were subsequently blocked in 5% skim milk. The blocking buffer was then removed, and the membranes were washed with TBST for incubation with primary antibodies at 4°C overnight. The membranes were washed three times with TBST and incubated with horseradish peroxidase-conjugated anti-mouse or anti-rabbit IgG secondary antibodies (31430, 31460; Thermo Fisher Scientific) for 1 h at room temperature. The secondary antibody was removed, and the membranes were washed three times with TBST. The membranes were incubated with West Femto Maximum Sensitivity Substrate (Thermo Fisher Scientific) for the detection of immunoreactive bands. ImageJ software was used for protein signal level quantification. Beta-actin was used as the loading control. The data were graphed and statistically tested using GraphPad Prism 9 and R 4.1.3. The experiment was repeated independently three times with similar results and representative images are shown. The primary antibodies used in this study were as follows: DPP3 (PA5-21709; Thermo Fisher Scientific), FASN (10624-2-AP; Proteintech, Rosemont, USA), and beta-actin (81115-1-RR; Proteintech).

### RNA isolation and quantitative real-time PCR

Total RNA was extracted from breast cancer tissues and adjacent normal tissues with TRIzol reagent (Invitrogen, Carlsbad, USA). Reverse transcription for cDNA synthesis was performed using the MonScript RTIII Super Mix with dsDNase kit (Monad Biotech, Suzhou, China). qPCR was carried out with MonAmp ChemoHS qPCR Mix (Monad Biotech) on a CFX Connect real-time PCR system (Bio-Rad Laboratories, Hercules, USA). Bio-Rad CFX Manager version 3.1 was used to collect real-time PCR data. The relative mRNA level was calculated via the 2 ^‒ΔΔCt^ method. *GAPDH* was used as the internal reference. The sequences of primers were as follows: *DPP3*, forward, 5′-CTACGTGAACTGGCTCAACATGG-3′, and reverse 5′-CTCCAGCAAGACTCTCAGGATC-3′; *FASN*, forward, 5′-TTCTACGGCTCCACGCTCTTCC-3′ and reverse, 5′-GAAGAGTCTTCGTCAGCCAGGA-3′; *GAPDH*, forward, 5′-GTCTCCTCTGACTTCAACAGCG-3′ and reverse, 5′-ACCACCCTGTTGCTGTAGCCAA-3′.

### Colony formation and CCK8 assays

For the colony formation assay, cells were seeded in 6-well plates (10 ^3^ cells/well). After 7 days, the cells were stained with crystal violet solution and the number of colonies were counted under a microscope.

A Cell Counting Kit-8 (CCK8; Yeasen Biotechnology, Shanghai, China) was used for cell viability analysis. Cells were seeded in a 96-well microplate and cultured at a density of 5×10 ^3^ cells/well in 100 μL of medium. Ten microliters of CCK8 reagent was added to each well and incubated for 2 h. The absorbance was measured at 450 nm using a microplate reader (BioTek, Winooski, USA), with wells without cells as blanks. All experiments were performed in triplicate.

### Wound healing assay

For the cell migration assay, cells were uniformly seeded in six-well plates and incubated until the cell density reached 95%. Then, the cell layers were scratched along the underside with a pipette tip. The cells were gently washed with PBS, and then a certain volume of serum-free medium was added. The cells were observed under a microscope and photographed according to the manufacturer’s instructions.

### Transwell assays

Matrigel (Sigma Aldrich, St Louis, USA) and medium were mixed at a ratio of 1:8~1:10. Then, 60‒100 μL of the mixture was spread evenly to the lower chamber of transwell plates (Corning, New York, USA) and incubated in the incubator for 2 h until the gel formed. Then, 600‒800 μL of medium supplemented with 10% FBS was added to the lower chamber. After cell counting, 20,000 cells in 200 μL of serum-free medium were added to the upper chamber. After 24 and 48 h, the lower chamber was gently cleaned with PBS and fixed with paraformaldehyde for 15 min. Then, the cells inside the filter membrane were stained with 0.1% crystal violet for 15 min. The residual dye was removed with PBS, and the sections were dried and observed under a microscope. The average cell number per well was calculated with ImageJ (NIH, Bethesda, USA).

### Cell cycle and apoptosis analysis

Transfected cells were collected and fixed with 70% ethanol for cell cycle analysis. The cells were then treated with RNase A and propidium iodide (PI) for 30 min and analyzed by flow cytometry. Transfected cells were collected after treatment with trypsin and then stained with Annexin V-FITC and PI (Beyotime) for apoptosis analysis. The cells were also analyzed by flow cytometry.

### Protein degradation assays

Cells stably expressing the indicated vectors in 6-well plates were allowed to reach approximately 70% confluence before being treated with the protein synthesis inhibitor cycloheximide (CHX, 100 μg/mL; MedChemExpress, Monmouth Junction, USA) or the protease inhibitor MG132 (20 μM; MedChemExpress) for the indicated time intervals. The cells were then collected and analyzed by western blot analysis with indicated antibodies.

### Immunoprecipitation assay

For immunoprecipitation, cells were transfected with the indicated plasmids and lysed in 1 mL of NP-40 buffer. Then, 0.9 mL of cell lysate was added to the indicated beads and incubated at 4°C overnight. The beads were collected by centrifugation and washed three times with NP-40 buffer. The immune complex was detected by western blot analysis with specific antibodies.

### Ubiquitination assay

For *in vitro* ubiquitination assay, cells were transfected with the indicated plasmids. Forty-eight hours later, the cells were treated with 20 μM MG132 for 5 h before being harvested. Cells were lysed in 1 mL of RIPA buffer and denatured by boiling for 5 min. Immunoprecipitation and western blot analysis were performed as described above.

### SRS imaging of breast cancer cells

MDA-MB-231 cells were plated in 35-mm glass-bottom dishes at 10 ^5^ cells/dish. Before SRS imaging, MDA-MB-231 cells were washed with PBS once. Subsequently, the MDA-MB-231 cells were fixed with 10% neutral formalin (Sigma-Aldrich, St Louis, USA) for 15 min. Finally, the MDA-MB-231 cells were washed with PBS and immersed in PBS for SRS imaging. SRS imaging was performed on a picosecond SRS microscope [16]. An ultrafast laser system with dual output at 80 MHz (picoEmerald; Applied Physics & Electronics, Berlin, Germany) provided both pump (tunable wavelength 700‒990 nm) and Stokes beams (fixed wavelength 1031 nm). The Stokes beam was modulated at 20 MHz by an electro-optic modulator. A 60× water objective (numerical aperture 1.2; Olympus, Tokyo, Japan) was used to focus the light into the sample.

### *In situ* LD analysis of the hSRS image stack

Phasor analysis was used to segment LDs (lipid droplets) from MDA-MB-231 cells based on the SRS spectral profile. In previous reports, the peak at 2750‒3050 cm ^–1^ was well fitted as the sum of seven Lorentzian bands [ 17, 18]. The Lorentz peak function was used for multi-peak fitting in OriginPro 2021 according to the fitting parameters listed in Supplementary Table S2. The maximum height of the Lorentz peak at ~3015 cm ^–1^ was used to quantify the unsaturated lipid level relative to the total lipid content. The total unsaturated lipid level is quantified by the product of the unsaturated lipid level relative to the total lipid in the normalized SRS spectrum of the lipid-rich region multiplied by the total lipid level in the LD region.

### Oil red O staining

Oil red O (0.5 g) was dissolved in 100 mL of isopropyl alcohol as a stock solution and diluted 3:2 with distilled water prior to use. After discarding the medium, methanol fixative was added and incubated for 5 min, and then the oil red O dye solution was added and incubated for 15 min. The samples were rinsed with water three times. Then, the color was separated by 60% ethanol, and the morphology and density of the lipid droplets were observed under a microscope (Joel Ltd, Tokyo, Japan).

### Mass spectrometry

Coomassie-stained SDS-PAGE gels containing DPP3 were destained with a solution of 50 mM ammonium bicarbonate in 50% acetonitrile (1:1, vol/vol) and reduced with 10 mM DTT at 56°C for 60 min, followed by alkylation with 55 mM iodoacetamide at room temperature for 45 min in the dark. The gel pieces were dehydrated in acetonitrile, rehydrated in 10 mM ammonium bicarbonate and treated with trypsin (Promega, Madison, USA) at an enzyme/substrate ratio of 1:50 (w/w) at 37°C overnight. Peptides were extracted from the gel pieces with 50% acetonitrile in 5% formic acid. An analytical column (75 μm inner diameter) was prepared for sample loading. The dried samples were resuspended in 10 μl of LC‒MS buffer A (0.1% FA), 5 μl of sample was transferred to autosampler vials, and then 1 μl of sample was injected onto an Easy-nano LC system (Thermo Scientific) coupled online with a Q Exactive HF mass spectrometer (Thermo Scientific). For MS/MS, the top 12 most abundant ions were automatically selected in each MS scan and fragmented in HCD mode. The LC‒MS/MS data were processed and searched against the human protein database downloaded from the UniProt database using Proteome Discoverer (version 2.5) for protein identification. The MS and MS/MS results were searched with a peptide ion mass tolerance of 10 ppm and a fragment ion mass tolerance of 0.02 Da.

### Untargeted metabolomics and liquid chromatography mass spectrometry (LC-MS/MS)

The untargeted metabolomics assay was performed by MetWare (Wuhan, China). WT and *DPP3*-KO MDA-MB-231 cells were collected and immediately frozen in liquid nitrogen. Each sample contained 10 ^7^ cells. The samples stored at –80°C were thawed on ice. Then, 500 μL of solution (methanol:water=4:1, V/V) containing an internal standard was added to the cell sample and vortexed for 3 min. The sample was placed in liquid nitrogen for 5 min and on dry ice for 5 min and then thawed on ice and vortexed for 2 min. This freeze‒thaw cycle was repeated three times. After centrifugation, two hundred microliter aliquots of the supernatant were used for LC-MS analysis.

All samples were subjected to two LC/MS methods. One aliquot was analyzed under positive ion conditions and eluted from a T3 column (Waters ACQUITY Premier HSS T3 Column 1.8 μm, 2.1 mm×100 mm) using 0.1% formic acid in water as solvent A and 0.1% formic acid in acetonitrile as solvent B. The data were acquired in information-dependent acquisition (IDA) mode using Analyst TF 1.7.1 Software (Sciex, Concord, Canada). The “SVR” method was used to correct the peak area. For two-group analysis, differentially abundant metabolites were determined by VIP (VIP>1) and *P* value ( *P* value<0.05, Student’s *t* test). To avoid overfitting, a permutation test (200 permutations) was performed. KEGG annotation and enrichment analysis identified metabolites were annotated using the KEGG Compound database ( http://www.kegg.jp/kegg/compound/), and the annotated metabolites were subsequently mapped to the KEGG Pathway database ( http://www.kegg.jp/kegg/pathway.html). Significantly enriched pathways were identified with a hypergeometric test *P* value for a given list of metabolites. The metabolomics data are shown in Supplementary Table S3.

### Gene set enrichment analysis

Significantly differentially expressed genes from RNA sequencing of WT and *DPP3*KO MDA-MB-231 cells were analyzed using GSEA. Analysis was performed using GSEA software version 4.3.3 and MSigDB gene set collections ( https://www.gsea-msigdb.org/gsea/index.jsp).

### Network analysis

RNA-seq (112 normal and 1105 tumor) data, clinical information and gene copy number variation (CNV) data were downloaded from The Cancer Genome Atlas (TCGA) database ( https://portal.gdc.cancer.gov/). The collected clinicopathological data included sex, age, stage, TNM classification, survival status and survival duration. Kaplan–Meier survival analysis of DPP3 in breast cancer patients was obtained from the Kaplan-Meier Plotter [19] ( http://kmplot.com/analysis/index.php?p=background). Cancer Cell Line Encyclopedia (CCLE) Affy and CNV data of cancer cell lines were downloaded from CCLE ( http://www.broadinstitute.org/ccle). Protein expression data were obtained from the Clinical Proteomic Tumor Analysis Consortium (CPTAC) database ( https://pdc.cancer.gov/pdc/). The Human Protein Atlas (HPA) database ( https://www.proteinatlas.org/) was used to determine the protein expression of DPP3 and FASN in individual tumor samples.

### Statistical analysis

For differential expression analysis, we estimated differentially expressed DPP3 between tumor and normal breast cancer samples from the TCGA. The fold change (FC) and the *P* values based on the Mann–Whitney test were considered indicators of dysregulation. Two-tailed Student’s *t* tests were used for comparisons of means of data between two groups. For multiple independent groups, one-way or two-way ANOVA with Tukey *post hoc* tests was used. *P*<0.05 was considered statistically significant. Other quantitative data are presented as the mean±standard deviation (SD). Pearson correlation analysis was employed to analyze the strength and direction of the linear relationship between two variables, represented as the R ^2^ value (square of the Pearson correlation coefficient R).

## Results

### DPP3 is upregulated in breast cancer and indicates poor prognosis

**Figure 1:**
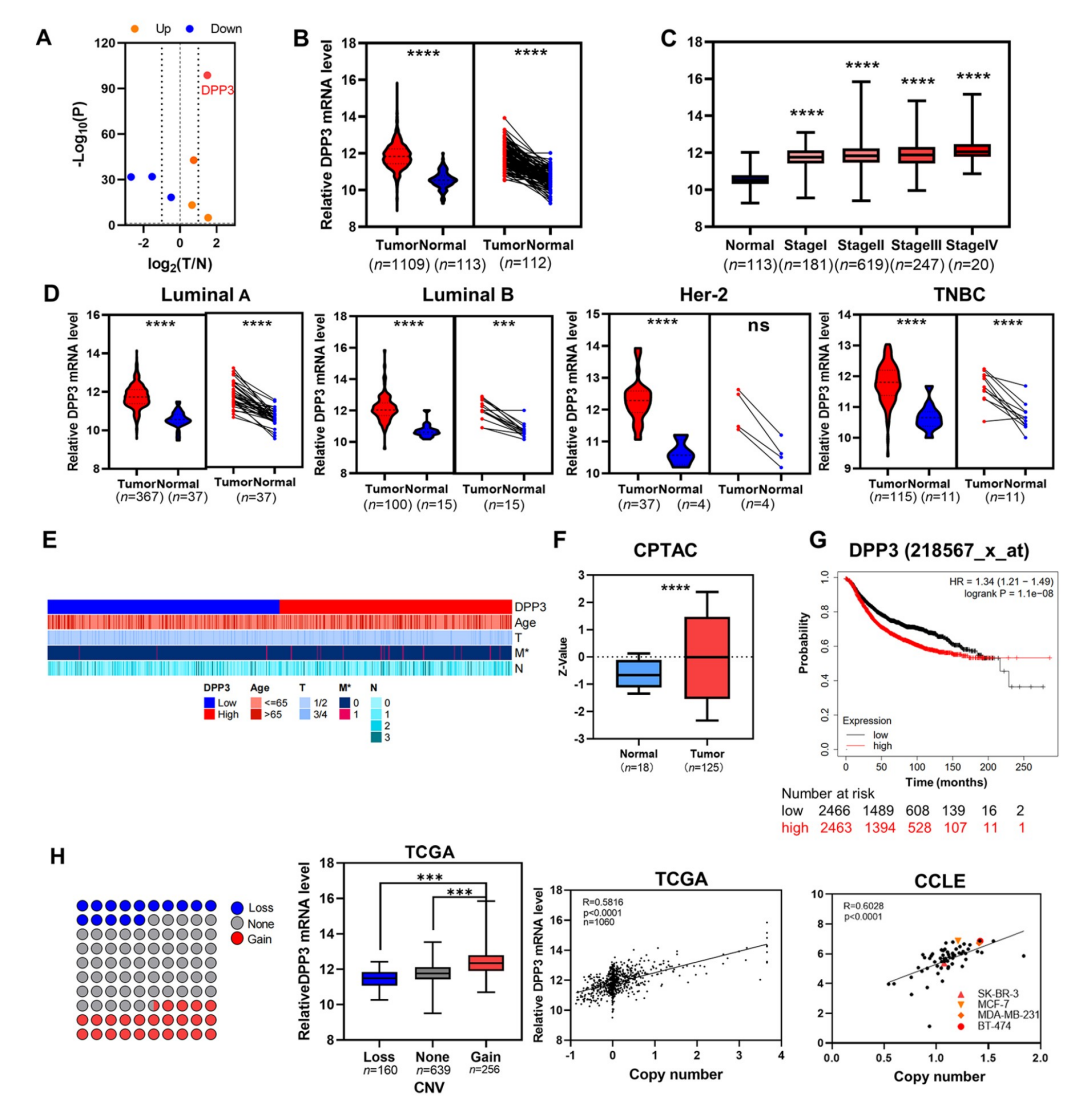
Figure 1 DPP3 mRNA and protein levels in breast cancer tissues are significantly increased and correlated with poor prognosis (A) Volcano plot of the expressions of 7 DPPs in breast cancer based on TCGA RNA-Seq data. DPP3 exhibits significant differential expression (P<0.001, absolute fold change>1.5) when comparing primary tumors with adjacent normal breast tissues (T/N). (B) Compared to those in unpaired or paired normal breast tissues, relative DPP3 mRNA levels are upregulated in breast tumors from the TCGA database. (C) The expression levels of DPP3 in breast cancer tissues at different stages are higher than those in normal tissues. (D) TCGA data were analyzed for the following breast cancer molecular subtypes: luminal A, luminal B, HER2+, and triple-negative breast cancer (TNBC). (E) The expression of DPP3 and corresponding clinical indicators in breast cancer samples in TCGA database. (F) DPP3 protein is highly expressed in breast cancer compared with that from the Clinical Proteomic Tumor Analysis Consortium (CPTAC) database. (G) Kaplan-Meier curves showing the overall survival of breast cancer patients based on DPP3 expression in breast cancer tissues from the TCGA database. (H) DPP3 mRNA levels and CNVs were assessed in the TCGA and CCLE Affy databases. R, Spearman correlation coefficient. Boxplots show the median, quartiles, min, and max. P values of the boxplots are based on the Mann-Whitney test. ***P<0.001, ****P<0.0001. ns, no significance.

Among the seven members of the DPP family, DPP3 exhibits the most significant difference in expression between the tumor-to-normal stage based on the breast cancer transcriptomic profile from the TCGA database ( Figure 1A). DPP3 is significantly more abundant in breast cancer tissues than in adjacent normal tissues regardless of the TNM stage and molecular subtype of breast cancer ( Figure 1B‒D). Based on the analysis of the distribution of DPP3 expression among clinicopathological features of breast cancer, upregulation of DPP3 is more common in patients with distant metastasis ( Figure 1E). Consistently, the DPP3 protein level is significantly higher in breast cancer tissues than in normal controls based on analysis of the CPTAC database ( Figure 1F). Kaplan-Meier curve analysis showed upregulation of DPP3 consistently correlates with significantly shorter overall survival in breast cancer patients ( *P*<0.0001; Figure 1G). To identify the cause of the upregulation of DDP3, we analyzed the relationship between copy number variation (CNV) and mRNA expression in the TCGA and CCLE Affy datasets. We found that the CNV of DPP3 is correlated with the upregulation of DPP3 mRNA. ( Figure 1H).

### DPP3 promotes the proliferation, migration and invasion of breast cancer cells

**Figure 2:**
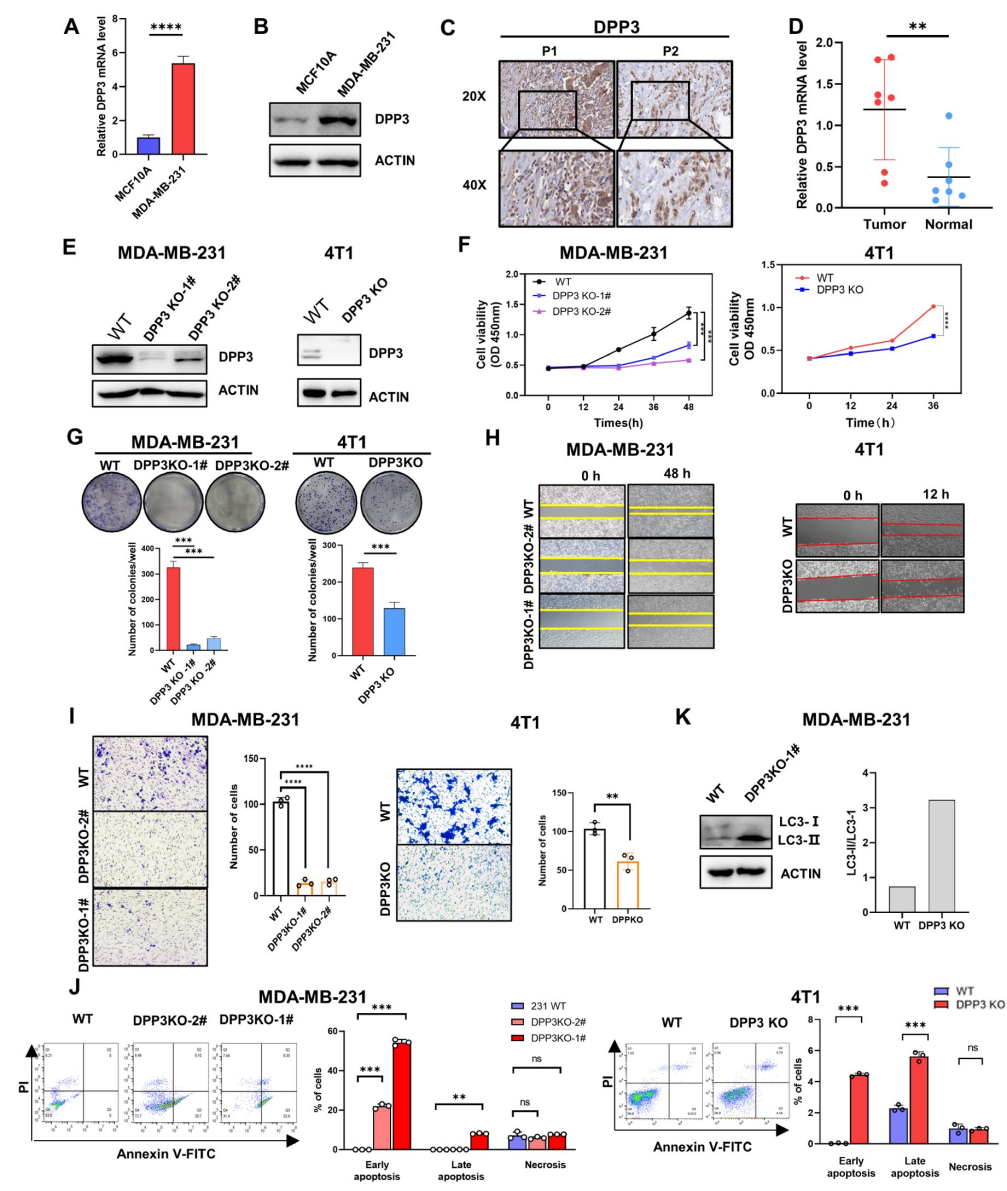
Figure 2 DPP3 promotes breast cancer tumorigenesis (A,B) qRT-PCR and western blot analysis results showed increased DPP3 mRNA (A) and protein (B) levels in MDA-MB-231 cells. (C) Immunohistochemical analysis of human breast cancer tissues showed that DPP3 was highly expressed in breast cancer tissues. (D) The mRNA levels of DPP3 in human breast cancer samples were determined by qRT-PCR. GAPDH was used as an internal reference gene. (E) Western blot analysis results showed the efficiency of DPP3 knockout by CRISPR Cas9 in the MDA-MB-231 (left) and 4T1 (right) cell lines. β-Actin was used as a loading control. (F,G) CCK-8 (F) and colony formation assays (G) showed that DPP3 KO significantly inhibited the proliferation of MDA-MB-231 (left) and 4T1 (right) cells. (H,I) Wound healing (H) and transwell (I) assays showed that DPP3 KO significantly decreased the invasion and migration ability of MDA-MB-231 (left) and 4T1 (right) cells. (J) Flow cytometry was used to detect the ratio of apoptosis in WT and DPP3-KO MDA-MB-231 (left) and 4T1 (right) cells stained with Annexin V-FITC and PI. Data are shown as the mean±SD. (K) Compared with that in the WT group, the LC3-II/LC3-I ratio in the DPP3KO group was greater than 1, suggesting that the loss of DPP3 may promote the occurrence of tumor autophagy. *P<0.05, **P<0.01, ***P<0.001, ****P<0.0001. ns, no significance.

We conducted a series of *in vitro* cell biology assays to explore the biological function and mechanisms of DPP3 in breast cancer. First, the mRNA and protein levels of DPP3 in MDA-MB-231 cells were found to be significantly higher than those in MCF10A cells ( Figure 2A,B). Additionally, DPP3 protein ( Figure 2C) and mRNA ( Figure 2D) were highly expressed in breast cancer tissues compared with those in adjacent breast tissues. CRISPR/Cas9 technology was used to construct MDA-MB-231 and 4T1 cell lines with stable *DPP3* knockout (KO) ( Figure 2E). CCK8 assays revealed that the proliferation abilities of MDA-MB-231 and 4T1 cells were impeded following *DPP3* KO ( Figure 2F). Figure 2G showed that colony formation was inhibited in stable *DPP3*-KO tumor cells. Next, the results of wound healing and transwell assays demonstrated that *DPP3* KO inhibited the migration and invasion abilities of tumor cells ( Figure 2H–I). Subsequently, flow cytometry assay showed that *DPP3* KO promoted tumor cell apoptosis ( Figure 2J). In addition, we explored whether DPP3 impacts the proliferation of cancer cells through autophagy. The autophagy marker LC3-Ⅱ/LC3-Ⅰ ratio increased in *DPP3*-KO MDA-MB-231 cells, suggesting that the loss of DPP3 might enhance autophagy in tumor cells ( Figure 2K). Collectively, these results revealed the effect of DPP3 on the proliferation, invasion, migration, apoptosis and autophagy of breast cancer cells.

### DPP3 stabilizes FASN expression

**Figure 3:**
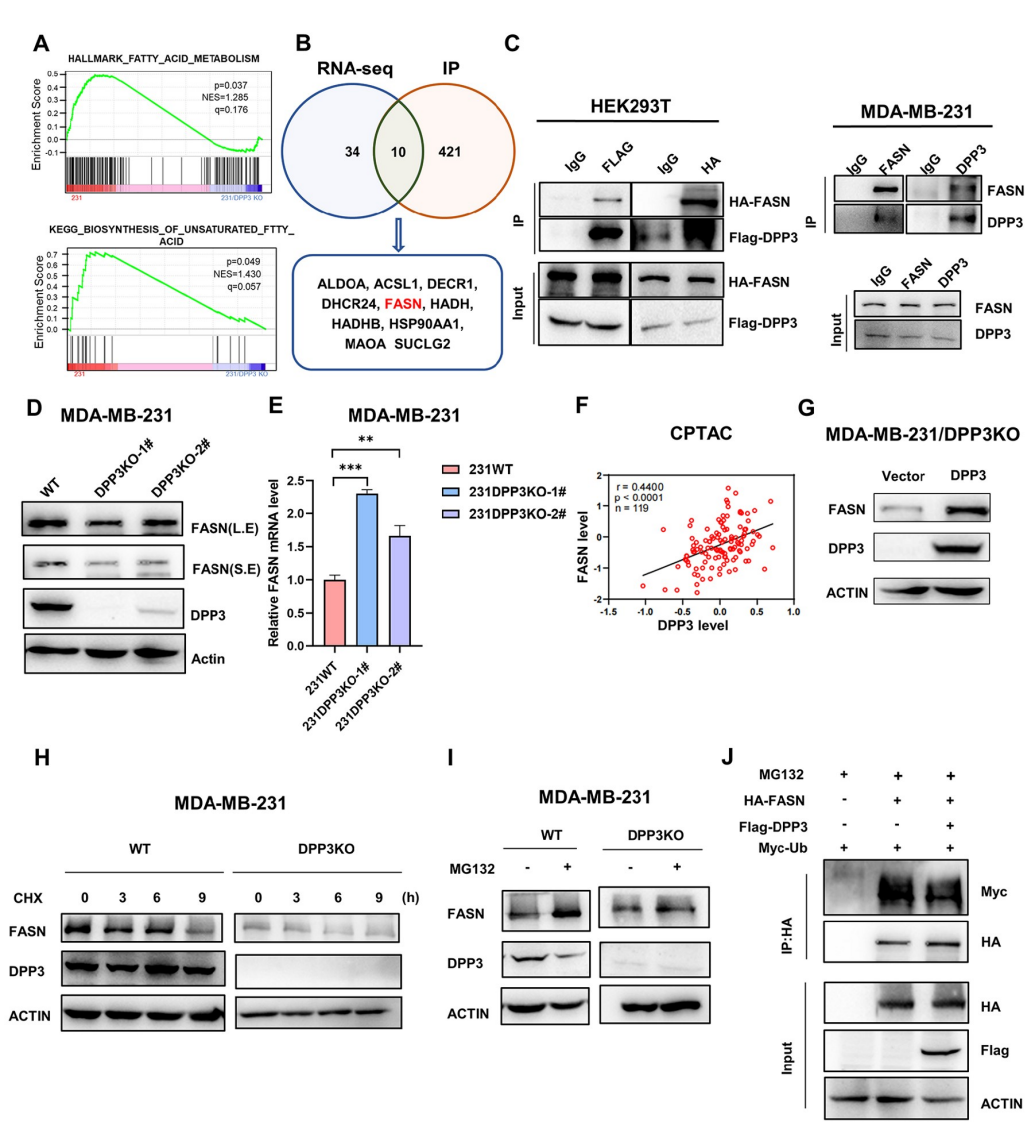
Figure 3 DPP3 stabilizes FASN expression (A) The differential genes enrichment analysis of the WT and DPP3KO groups showed that FASN was enriched in fatty acid metabolic pathway and biosynthesis of unsaturated fatty acid pathway. (B) Flow chart for selecting FASN by RNA sequencing in WT and DPP3-KO MDA-MB-231 cells and IP mass spectrometry. (C) FASN was identified as a DPP3-interacting protein. Left: Flag-DPP3 WT and HA-FASN were co-expressed in HEK293T cells for 48 h. Right: Co-IP experiments of endogenous DPP3 and FASN were performed in MDA-MB-231 cells. (D) Western blot analysis showed that FASN expression was significantly reduced in MDA-MB-231 cells with DPP3 knockout compared to that in WT cells. β-Actin was used as a loading control. (E) qRT-PCR showed that FASN mRNA expression was significantly increased in MDA-MB-231 cells with DPP3 knockout compared to that in WT cells. GAPDH was used as a reference gene. (F) The correlation of the protein expression of DPP3 and FASN was obtained from the Clinical Proteomic Tumor Analysis Consortium (CPTAC) database. (G) Restoration of DPP3 in DPP3-KO MDA-MB-231 cells restored FASN expression. (H) Interaction between endogenous DPP3 and FASN after treatment with the protein synthesis inhibitor CHX for 9 h before collection. The expression of the target protein in the lysate was detected by western blot analysis. (I) Western blot analysis showed that FASN protein expression increased significantly after MG132 treatment for 6 h in WT and DPP3-KO MDA-MB-231 cells. (J) In vivo polyubiquitination of FASN in the presence or absence of DPP3. HEK293T cells were transfected with the indicated plasmids for 48 h, followed by MG132 treatment for 5 h before lysis. Immunoprecipitation of ubiquitin-conjugated FASN proteins was performed with anti-HA magnetic beads. Immuno-complexes and lysates (input) were analyzed by western blot analysis using the indicated antibodies.

Next, GSEA analysis of the differentially expressed proteins showed that DPP3 significantly affects the lipid fatty acid metabolism pathway and unsaturated fatty acid synthesis pathway ( Figure 3A). To systematically identify the direct and key regulators of DPP3, differentially expressed proteins and coimmunoprecipitation (Co-IP) proteins that interact with DPP3 between WT and *DPP3*-KO MDA-MB-231 cells were identified via SDS-PAGE combined with mass spectrometry. According to the RNA sequencing results for the WT and *DPP3*-KO MDA-MB-231 cells, 44 differential genes (DEGs) involved in the fatty acid metabolism pathway were screened, with *P*<0.01 and FC ratios greater than 1.3 or less than 0.769. According to the results of IP mass spectrometry, 10 of these 44 DEGs were found to bind with DPP3 ( Figure 3B). Among them, a key enzyme for fatty acid synthesis, FASN, was identified as a protein that interacts with DPP3 based on mass spectrometry (mass spectrometry results in Supplementary Table S4). Therefore, we speculated that DPP3 might promote tumor progression by regulating FASN. Co-IP assays also showed that both exogenous and endogenous DPP3 interacted with FASN ( Figure 3C). Moreover, western blot analysis showed that FASN protein expression was significantly repressed following *DPP3* KO ( Figure 3D), while FASN mRNA level was increased ( Figure 3D). This finding suggested that DPP3 might regulate FASN expression through its direct interaction with FASN protein. The coexpression of DPP3 and FASN proteins was also validated based on the CPTAC database. The FASN protein level is positively correlated with the DPP3 protein level ( Figure 3F). In addition, the re-expression of DPP3 in *DPP3*-KO MDA-MB-231 cells restored the expression of FASN protein ( Figure 3G). We speculated that DPP3 may affect the stability of FASN. After protein synthesis was blocked by CHX, we found that the loss of DPP3 shortened the half-life of endogenous FASN in MDA-MB-231 cells ( Figure 3H). The protease inhibitor MG132 significantly inhibited FASN protein degradation in both WT and *DPP3*-KO MDA-MB-231 cells ( Figure 3I). These findings revealed that DPP3 can stabilize FASN by preventing the degradation of FASN proteasome pathway. The degradation efficiency of the proteasome pathway is mainly regulated by the ubiquitination and deubiquitination pathways. Therefore, Myc-Ub was transfected into HEK293T cells to observe FASN ubiquitination, and we found that simultaneous transfection of HA-FASN and Flag-DPP3 promoted FASN ubiquitination ( Figure 3J). Collectively, these results confirmed that DPP3 can stabilize FASN expression.

### DPP3 affects the synthesis of fatty acids

**Figure 4:**
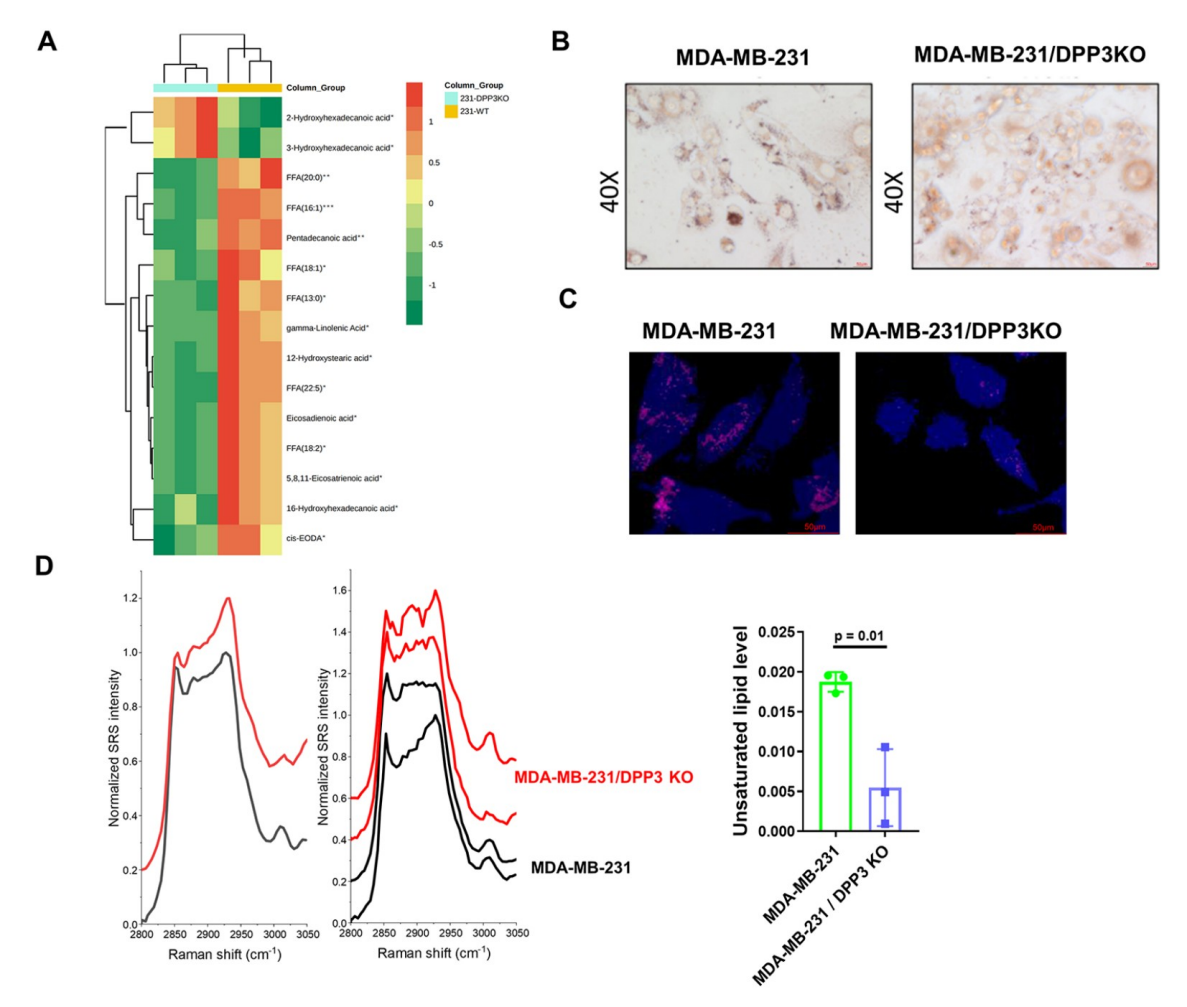
Figure 4 DPP3 affects the metabolism of fatty acids MDA-MB-231 cells with or without DPP3 knockout were subjected to the following experiments. (A) Metabolomic analysis of fatty acids in the WT and DPP3-KO MDA-MB-231 cells. The samples were measured by gas chromatography-mass spectrometry (GC-MS) (n=3). (B) Oil red O staining of cells. (C) The LD region and protein-rich region were segmented by spectral phasor analysis, where blue represents the protein-rich region and magenta represents the lipid-rich region. (D) Normalized SRS spectrum of the lipid droplet (LD) region and its unsaturated lipid level in MDA-MB-231 cells.

Next, we sought to determine the effect of DPP3 on fatty acid synthesis. Metabolomics analyses of WT and *DPP3*-KO MDA-MB-231 cell lines were performed by LC-MS/MS, which revealed that DPP3 deletion resulted in a significant reduction in FFA(18:1), 12-hydroxystearic acid, FFA(13:0), FFA(18:2), eicosadienoic acid, FFA(20:0), gamma-linolenic acid, 16-hydroxyhexadecanoic acid, FFA(16:1), FFA(22:5), cis-EODA, 5,8,11-eicosatrienoic acid, and pentadecanoic acid, but a significant increase in 2-hydroxyhex adecanoic acid and 3-hydroxyhexadecanoic acid ( Figure 4A). Most of these fatty acids may participate in disease progression, and related studies are summarized in Table 1. We found that the MDA-MB-231 cells with *DPP3* KO had lower levels of unsaturated lipids and fewer lipid droplets than the MDA-MB-231 cells ( Figure 4B). To map the distribution of lipids with no intervention in cells, the representative segmentation results of LDs and protein-rich regions are shown in Figure 4C. We performed hSRS imaging in WT and *DPP3*-KO MDA-MB-231 cells. Normalized SRS spectra of the LD region are shown in Figure 4D. According to these results, a greater LD distribution can be observed. Through Lorentzian peak fitting, we quantified the total unsaturated lipid level, and the results showed that *DPP3* KO decreased unsaturated lipid accumulation in MDA-MB-231 cells.

**Table:** **Table 1** The studies on 15 screened differentially expressed fatty acids in different tumors

Compounds	Formula	Types of cancer	Types of experimental sample	Details
FFA(18:1)	C18H34O2	Breast cancer	Cell line	Increase phophatidylinositol 3-kinase (PI3-K) activity to stimulated cell proliferation [20].
			Cell line	Promote the secretion and invasion of MMP-9 in breast cancer cells [21].
			Cell line	Inhibit lipid peroxidation and ferroptosis in triple-negative breast cancer cells [22].
			Cell line	Mediate the production of arachidonic acid (AA), and then AA metabolites mediate FAK phosphorylation and cell migration [23].
		Lung carcinoma	Cell line	Prevent cancer formation through STAT phosphorylation with IFN-gamma [24].
		Tongue squamous cell carcinoma	Cell line	Induce apoptosis and autophagy by blocking the Akt/mTOR pathway [25].
		Hepatocellular carcinoma	Human	As the therapeutic target and biomarker [26].
		Colorectal cancer	Cell line and animal model	Oleic acid-induced NOX4 is dependent on ANGPTL4 expression to promote human colorectal cancer metastasis [27].
		Ovarian cancer	Cell line	Stimulate ovarian cancer cell proliferation by activating PPARα [28].
		Prostate cancer	Cell line	Promote the malignant phenotype of prostate cancer through G protein-coupled receptor FFA1/GPR40 [29].
		Malignant pleural effusions	Human	FFA 18:1-to-ceramide (d18:1/16:0) ratio as novel cancer biomarkers [30].
12-Hydroxystearic acid	C18H36O3	-
2-Hydroxyhexadecanoic acid	C16H32O3	-
FFA(13:0)	C13H26O2	-
FFA(18:2)	C18H32O2	Breast cancer	Cell line	Mediate migration and invasion through fascin [31].
			Cell line	To silence heat shock protein 90B1 (HSP90B1) to inhibit migration [32].
			Cell line	Induce an increased response to insulin [33].
			Cell line	Modulate the expression of breast cancer involved miRNAs [34].
			Cell line	Induce migration and invasion through FFAR4- and PI3K/Akt-dependent pathway [35].
		Non-small cell lung cancer	Human	A potential biomarker [36].
		Ovarian cancer	Cell line	Enhance cell migration by altering the dynamics of microtubules and the remodeling of the actin cytoskeleton at the leading edge [37].
		Colorectal cancer	Cell line	Induce cancer cell apoptosis by enhancing cellular oxidant status and inducing mitochondrial dysfunction [38].
			Cell line and animal model	Upregulate microrna-494 to induce quiescence [39].
Eicosadienoic acid	C20H36O2	Prostate cancer	Human	Early metabolic markers [40].
FFA(20:0)	C20H40O2	Colorectal cancer	Human	Positively associated with colorectal cancer risk [41].
		Neuroblastoma	Cell line	Reduce oxidative stress levels [42].
gamma-Linolenic acid	C18H30O2	Breast cancer	Cell line	Deplete Ca2+ stores and induces endoplasmic reticulum and oxidative stresses to cause death of breast cancer BT-474 cells [43].
			Cell line	Reduce the expression and secretion of SPARC [44].
			Cell line	Inhibit cell-wall synthesis by the curtailment of HIF-1α and FASN level [45].
			Cell line	Synergistic interaction between vinorelbine and gamma-linolenic acid [46].
			Cell line	A useful adjunct to primary tamoxifen [47].
		Gastric cancer	Human	Inhibit hypoxia-induced gastric cancer cell growth and epithelial-mesenchymal transformation by inhibiting the Wnt/b-catenin signaling pathway [48].
		Colon cancer	Cell line	Inhibit tumour-matrix adhesion via the inhibition of FAK and paxillin tyrosine phosphorylation [49].
		Non-small cell lung cancer	Cell line	Suppress hypoxia-induced proliferation and invasion by inhibition of HIF1α [50].
		Hepatocellular carcinoma	Animal model	Regulate PHD2 mediated hypoxia and mitochondrial apoptosis [51].
		Pancreatic cancer	Cell line	Synergistic effect with gemcitabine at concentrations that correspond to *in vivo* therapeutic doses [52].
16-Hydroxyhexadecanoic acid	C16H32O3	Pancreatic ductal adenocarcinoma	Human	A potential biomarker for the detection of resectable pancreatic ductal adenocarcinoma [53].
FFA(16:1)	C16H30O2	Breast cancer	Cell line and animal model	Palmitoleic acid in blood, promote ACSL4-dependent tumor ferroptosis induced by IFNγ plus arachidonic acid [54].
		Ovarian cancer	Human	The percentage of palmitoleic acid in peritoneal fluid of cancer patients was significantly increased [55].
		Colorectal cancer	Human	The content of palmitoleic acid in colorectal cancer tissues decreased significantly [56].
		Prostate cancer	Human	Palmitoleic acid is significantly elevated in cancer patients [57].
FFA(22:5)	C22H34O2	-
cis-EODA	C18H34O3	-
3-Hydroxyhexadecanoic acid	C16H32O3	-
5,8,11-Eicosatrienoic acid	C20H34O2	Breast cancer	Cell line	The suppression of breast cancer cell growth and metastasis were examined *in vitro* and *in vivo* by using the KPL-1 human breast cancer cell line [58].
			Animal model	May be a beneficial chemotherapeutic agent for the luminal A subtype of breast cancer [59].
		Squamous cell carcinoma	Cell line	Down regulator of antimetastatic E-cadherin and desmoglein expression [60].
Pentadecanoic acid	C15H30O2	Breast cancer	Cell line	To enhance the efficacy of tamoxifen endocrine therapy [61].
			Cell line	Suppress interleukin-6 (IL-6)-induced JAK2/STAT3 signaling, and promoted caspase-dependent apoptosis in MCF-7/SC [62].
			Human	The cytidine-5-monophosphate/pentadecanoic acid metabolic ratio was the most significant discriminator between cancer and normal tissues [63].
		Colorectal cancer	Human	Identified as a significant negative association with colorectal cancer risk [64].

### DPP3/FASN expression in breast cancer patient samples

**Figure 5:**
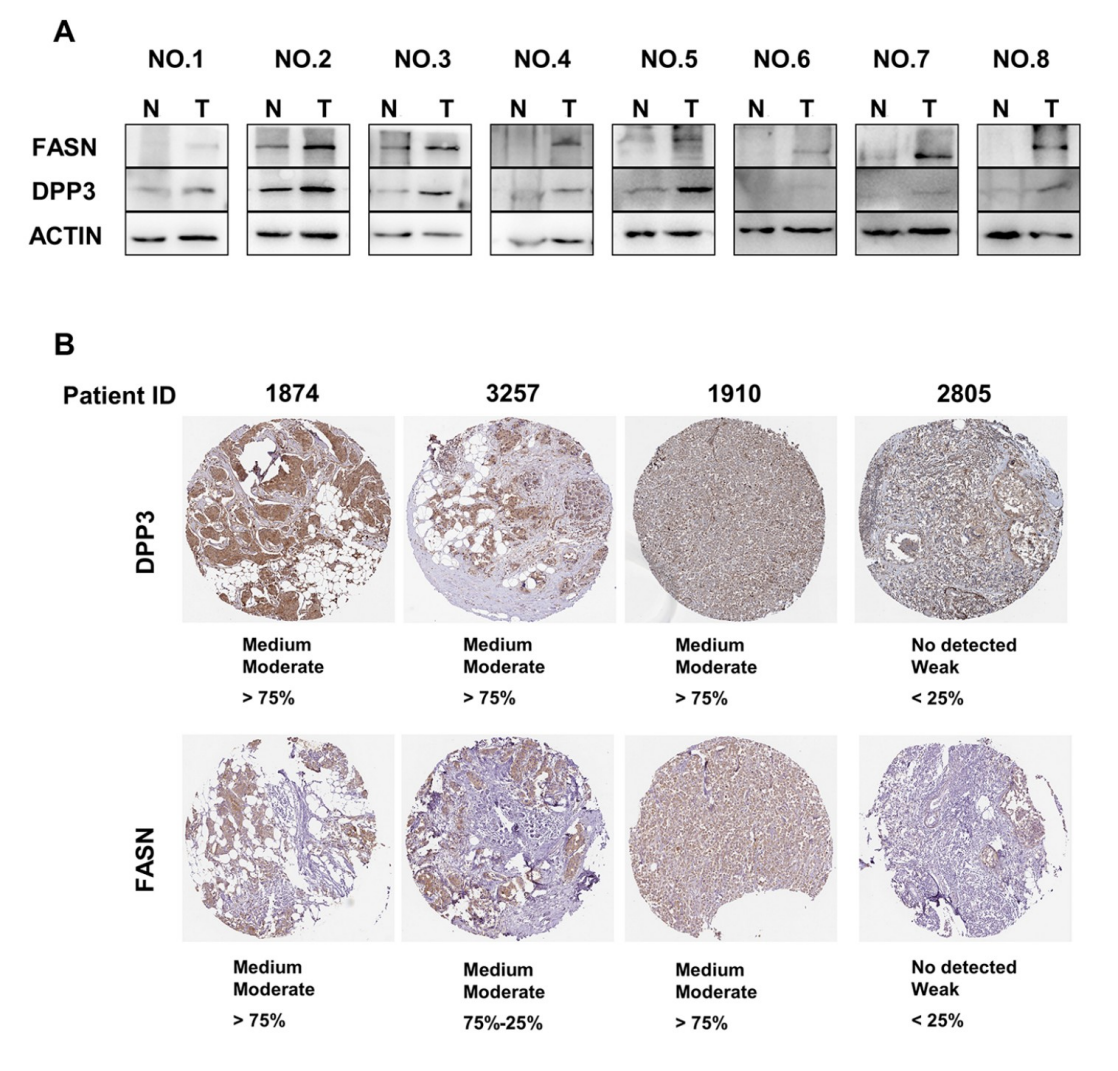
Figure 5 The expressions of DPP3 and FASN in breast cancer patients (A) The protein levels of DPP3 and FASN in human breast cancer samples were evaluated by western blot analysis. β-Actin was used as a loading control. (B) The protein expressions of DPP3 and FASN in individual tumor samples from the HPA database.

Western blot analysis of human breast cancer samples also showed that DPP3 and FASN were more highly expressed in breast cancer tissues than in normal tissues ( Figure 5A). Immunohistochemistry results showed that the expression of DPP3 in individual tumor sample from the HPA database was positively correlated with FASN ( Figure 5B). The above analysis in BC patient samples suggested that DPP3 might have the potential function related to the stability of FASN and promotes the occurrence and development of breast cancer.

## Discussion

Breast cancer is the most common cancer among women worldwide [1]. Due to the tumor heterogeneity, identifying the key regulator required for the development of breast cancer and its behavior has important implications for making optimal treatment decisions. Unlike its family member DPP4, the role of DPP3 in tumor biology is much less clear [ 10, 65, 66]. Emerging studies have shown that DPP3 expression is upregulated in some types of cancers [ 5‒ 8]. In esophageal cancer, overexpression of DPP3 promotes tumor growth and indicates poor prognosis [67]. Our study suggested that DPP3 is also highly correlated with poor prognosis in breast cancer patients. And the upregulation of DPP3 may be driven by increased DNA copy number. Based on our results, DPP3 might be a novel prognostic biomarker for breast cancer. Lu *et al*. [9] found that DPP3 could support breast cancer cell survival by stabilizing the expressions of other proteins. Our study focused on the role of the DPP3 protein in breast cancer. *DPP3* KO in breast cancer cells can significantly inhibit the growth and migration of tumor cells and induce apoptosis. These results suggest that DPP3 may be a new therapeutic target for breast cancer patients. There is still a long way to go from bench to bedside. Consequently, it is urgent to clarify its specific role and molecular mechanism in breast cancer.

Surprisingly, we found that DPP3 could interact with FASN and subsequently stabilize FASN. Many tumors rely on increased fatty acid synthesis to maintain robust cell proliferation. FASN serves as the rate-limiting enzyme in the FA synthesis pathway, which is directly linked to breast cancer, and its upregulation is associated with tumor aggressiveness [68]. Cell metabonomics showed that the stabilizing effect of DPP3 on FASN results in high levels of fatty acid synthesis. The levels of a series of essential fatty acids, such as oleic acid (FFA(18:1)), linoleic acid (FFA(18:2)), gamma-linolenic acid, palmitoleic acid (FFA(16:1)) and arachidic acid (FFA(20:0)), are significantly reduced in DPP3-deficient breast cancer cells. Among them, FFA(18:1) can stimulate cell proliferation by increasing the activity of phosphatidylinosiol 3-kinase (PI3-K) [20]. It can promote the secretion and invasion of MMP-9 in breast cancer cells [21]. Linoleic acid can also induce migration and invasion through FFAR4- and PI3K/Akt-dependent pathways [35]. The modification of the Wnt protein by palmitoleic acid is essential for the activation of the Wnt signaling pathway [69]. With regard to the response to therapy, pentadecanoic acid can enhance the efficacy of tamoxifen endocrine therapy. Vinorelbine has a synergistic effect with gamma-linolenic acid [46]. These results suggest that DPP3 may further affect the levels of fatty acids required for growth and proliferation via the regulation of FASN.

Nevertheless, there are some limitations in this study. First, this study was mainly carried out using breast cancer cell lines, and further studies are needed to investigate the effect of the DPP3/FASN axis on breast cancer progression *in vivo*. In addition, PDX models should be used to develop novel anti-DPP3 targeted therapies. Second, due to the small clinical sample size in real-world data, it was difficult to conduct stratified analysis to identify the association of DPP3 with several clinicopathological features. In addition, our results showed that DPP3 might be a potential target in the treatment of breast cancer, but the underlying molecular mechanism still needs to be further studied.

In summary, this study demonstrated the role of DPP3 in the reprogramming of fatty acid metabolism in tumors, and that upregulation of DPP3 driven by DNA copy number increase is associated with poor prognosis. This study revealed that DPP3-FASN interaction might be a new therapeutic target and a novel prognostic biomarker for breast cancer.

## Supporting information

STable_S2

STable_S3

STable_S1

STable_S4

## Supplementary Data

Supplementary data is available at *Acta Biochimica et Biophysica Sinica* online.
